# Real-Time Evaluation of the Signal Processing of sEMG Used in Limb Exoskeleton Rehabilitation System

**DOI:** 10.1155/2018/1391032

**Published:** 2018-10-14

**Authors:** Baofeng Gao, Chao Wei, Hongdao Ma, Shu Yang, Xu Ma, Songyuan Zhang

**Affiliations:** ^1^Key Laboratory of Convergence Medical Engineering System and Healthcare Technology, The Ministry of Industry and Information Technology, School of Life Science, Beijing Institute of Technology, No. 5, Zhongguancun South Street, Haidian District, Beijing 100081, China; ^2^Tianjin Key Laboratory for Control Theory & Applications in Complicated System, The School of Electrical and Electronics Engineering, Tianjin University of Technology, Tianjin, China; ^3^State Key Laboratory of Robotics and System, Harbin Institute of Technology, Harbin, China

## Abstract

As an important branch of medical robotics, a rehabilitation training robot for the hemiplegic upper limbs is a research hotspot of rehabilitation training. Based on the motion relearning program, rehabilitation technology, human anatomy, mechanics, computer science, robotics, and other fields of technology are covered. Based on an sEMG real-time training system for rehabilitation, the exoskeleton robot still has some problems that need to be solved in this field. Most of the existing rehabilitation exoskeleton robotic systems are heavy, and it is difficult to ensure the accuracy and real-time performance of sEMG signals. In this paper, we design a real-time training system for the upper limb exoskeleton robot based on the EMG signal. It has four main characteristics: light weight, portability, high precision, and low delay. This work includes the structure of the rehabilitation robotic system and the method of signal processing of the sEMG. An experiment on the accuracy and time delay of the sEMG signal processing has been done. In the experimental results, the recognition accuracy of the sEMG is 94%, and the average delay time is 300 ms, which meets the accuracy and real-time requirements.

## 1. Introduction

Stroke, also known as cerebrovascular accident (CVA), is a disease caused by acute cerebrovascular injury. Sequelae are often seen as loss of hemiplegia, facial paralysis, visual impairment, and language impairment. Hemiplegia is the most common symptom of a stroke [1]. According to the statistics of the Ministry of Health, cerebrovascular disease is the second cause of death in the population, and the surviving patients are left with various degrees of sequelae of hemiplegia, which brings a heavy burden to families and the society. Therefore, the treatment and rehabilitation of hemiplegic patients following cerebrovascular disease are particularly important and urgent, and the scholars in the related disciplines are also studying how to improve the rehabilitation effect. The rehabilitation therapy for hemiplegia after stroke which is proved to be an effective method has become a focal point of modern rehabilitation medicine and rehabilitation projects.

Functional compensation and reorganization of the human cerebral cortex are of great importance in the treatment of hemiplegic patients. However, this functional reorganization is also influenced by external environmental stimuli [2]. Clinical practice shows that external functional recovery can play an important role in all stages of neurological impairment. Through continuous movement and relearning, the plasticity of the nervous system has been strengthened and consolidated. Exercise relearning is a popular method in hemiplegic rehabilitation [3]. This method is based on neurophysiology, sports science, biomechanics, and behavioral science. It emphasizes the importance of the subjective participation and cognition of the patients. It also regards the recovery training of the motor function after the central nervous system injury as a process of exercise relearning. Thus, the rehabilitation therapy for hemiplegia after stroke which is proved to be an effective method has become a focal point of modern rehabilitation medicine and rehabilitation projects [4].

The development of an upper limb rehabilitation training robot started earlier, and its development in western countries has relatively matured. In 1991, researchers from MIT in the United States developed the world's first upper limb rehabilitation training robotic system, called MIT-MANUS. The robot adopted a connecting rod mechanism, through a handle at the end of the patient's hand. It can complete the motion in a two-dimensional plane, and the robot is connected to a computer. It can provide real-time visual feedback for the patient. In the process of sports training, parameters are collected and evaluated according to the parameter and evaluation system. In 2005, researchers at the University of Zurich developed a rehabilitation robot called ARMin, which has the characteristics of low inertia, low friction, and directional driving. It has four kinds of moving patterns, mainly in the four modes of rehabilitation training for patients. In the course of training, it can record the basic information of its movement track and then evaluate the whole training [5–8].

In the field of sEMG signal processing, the researchers have also made a lot of achievement. A. M. Simon and other scholars have identified more than ten kinds of action types of the upper limb and wrist. In this method, they select four time domain features to construct the feature space and use the LDA (linear discriminant analysis) method to identify the moving pattern, a method which finally achieved an accuracy of 86%–96%. In addition, in view of the nonstationarity of the sEMG signals, the scientific research team of Kazakhstan extracted the AR model parameters of an EMG signal to construct the feature space, then they used the method of principal component analysis (PCA) to reduce the dimension of the feature space, and lastly, they used the Back Propagation Neural Networks (BPNN) as the classification method [9, 10].

From the existing studies, we can see that the research trend for an upper limb exoskeleton robot is to make it portable, lightweight, and intelligentized. In this paper, we developed a system for an upper limb exoskeleton rehabilitation robot with 3 DOFs and where the control of robot motion is based on pattern recognition of the sEMG. In the signal processing of the sEMG, we extract the characteristics of the time and frequency signal to construct the feature space, and use random forest as the classifier for pattern recognition. In the final system test, we tested the classification accuracy of this classification result.

In this paper, some domestic and foreign researches about upper limb rehabilitation robots are introduced. Then, an upper limb exoskeleton rehabilitation robotic system is presented, and the experiments are carried out for the accuracy of its pattern recognition based on sEMG. The results are then discussed.

## 2. System Structure Design

### 2.1. Upper Limb Exoskeleton Design

Three key requirements should be considered. First of all, in order for patients to wear it, it should be flexible and light weight, and it should have high accuracy. Secondly, it should have the ability to apply enough power for combinatorial actions of the patients. Thirdly, the movement space must conform to the physiological structure [11].

To achieve the goal of our design, the device should provide enough torque to train the upper limb. The actuator should have high power to drive the device and move the upper limbs. The material of the device should have high strength to prevent the device from performing poorly in sports.

For most patients, the length or width of the upper limb has many dimensions and should be carefully considered. Therefore, when we design flexible, wearable, and adjustable concepts for most patients, the adjustable design is much better.

In addition, combined exercise is more beneficial to the rehabilitation process of patients'. Our devices should be free to carry out combined movements without interference. We designed a rehabilitation robot with three DOFs. Compared to the previous device, this design can overcome some shortcomings which makes it flexible, portable, light weight, highly accurate, and easy to wear [12].

**Figure 1:**
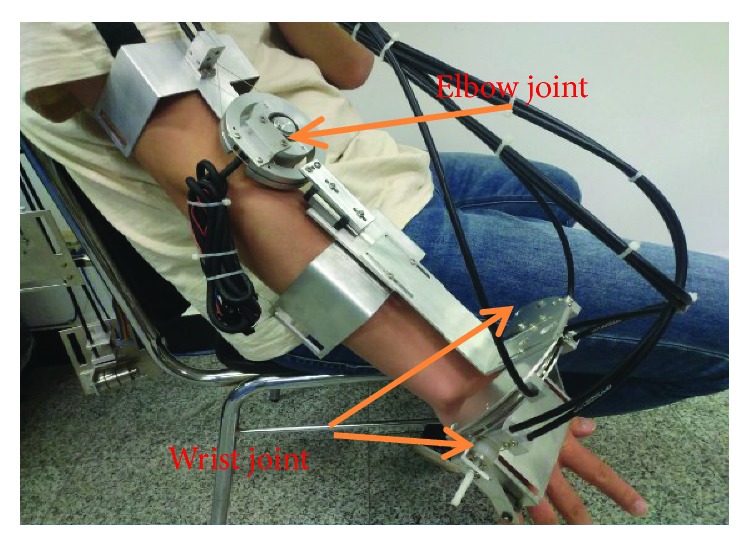
The structure of the upper limb exoskeleton.

The robot is shown in Figure 1. It has the following characteristics:

1. The stability of the wear. The upper limb rehabilitation robot has fixed grooves on the upper back, forearm, and upper arm of the human body, respectively, and is fixed with bandages in these places. These fixed places can guarantee the normal movement of 3 degrees of freedom.
2. Low weight. The common exoskeleton device is to put the motor on the axis of the arm movement, but the robot uses a rope driven method to remove the motor from the exoskeleton robot, greatly reducing its weight and reducing the burden of the patient [12].
3. Taking into account the size and range of motion of each person's upper limbs, many adaptive passive adjustment devices have been added to the robot to adapt to its changes. This also improves the comfort of the patient.

This robot has 3 parts, one for elbow joint and two for the wrist. The elbow joint of the human body has only one DOF, flexion and extension. The wrist has three DOFs, but we only designed for two DOFs, namely flexion and extension, as well as pronation and supination. The range of each motion is shown in Table 1 [13, 14].

### 2.2. Collection and Processing of sEMG

**Figure 2:**
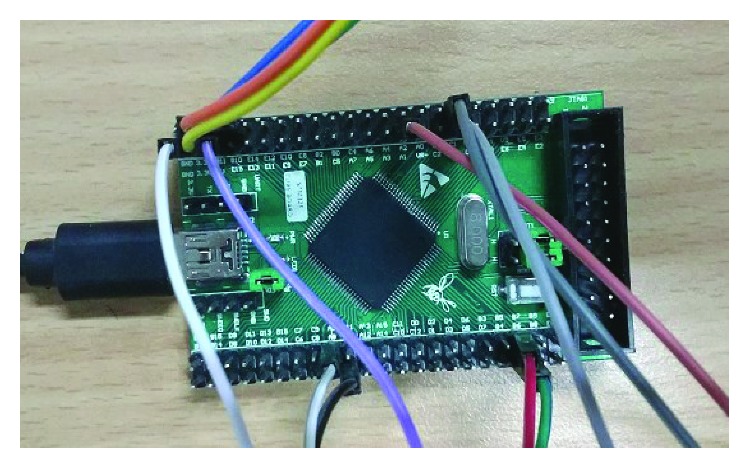
The sEMG acquisition instrument.

The exoskeleton robotic system has real-time recognition of sEMG signals and signal processing; therefore, the acquisition device uses real-time acquisition of EMG chips. As shown in Figure 2. The sEMG acquisition instrument can collect the original myoelectric signal and rectify and integrate on the basis of the original electromyographic signal, which is carried out in the chip. The output of the chip can be a rectified integral sEMG signal, and the most original surface EMG signal can also be the output. When electromyography is used to collect myoelectric signals, the position of the electrode sticking is very important. The sEMG acquisition instrument has two channels, and each channel has 2 electrode posts that are attached at the middle and ends of the measured muscles, respectively. There is also a muscle free part attached to the electrode.

The collected signal is sent as output to the digital multimeter, and the digital multimeters function as an AD conversion device. Then, the signal collected by the digital multimeter is connected to the computer and sent as output to the computer under the pretext of USB. We then use Matlab software to process signals and pattern recognition.

## 3. System Evaluation

### 3.1. Experimental Design and Data Collection

In the evaluation of the robot, we collect and process the EMG signals to get information, and then we control the movement of the robot in real time. Therefore, the purpose of the experimental design is to evaluate real-time and accurate acquisition and processing of electromyographic signals in the robotic system. In this process, EMG signal feature extraction and classifier design are two core issues.

In this experiment, we have designed the action mode of a BFNND elbow with four action modes: 0 degrees, 30 degrees, 60 degrees, and 90 degrees. We then collected the electromyographic signals of these four angles. The surface electromyographic signals of biceps brachii and triceps brachii muscle were collected by using a dual channel electromyographic acquisition device. 100 sets of data were collected and the duration of each group was 1 second. The frequency of EMG acquisition is 1000 Hz, so there are 1000 points in each group. In this way, 100 sets of dual channel data points were obtained.

**Figure 3:**
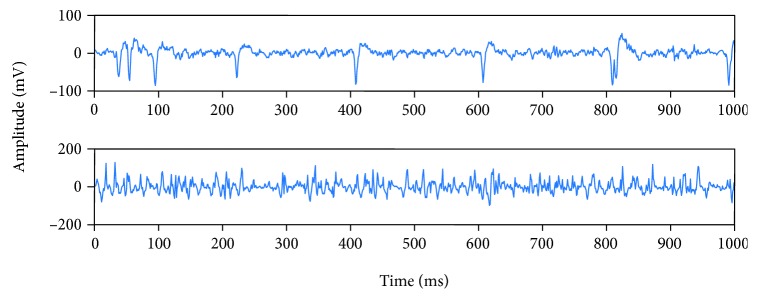
Time domain at a degree of 0.

Figure 3 shows the time domain information of 1 set of dual channel signals at the degree of 0. This is the original signal collected from the electromyographic acquisition instrument. After that, signal processing and pattern recognition will also be done.

### 3.2. Signal Preprocessing

According to the low frequency, weak, and nonlinear characteristics of the sEMG signal, we first carry on the band through an aluminum foil for the signal, and the bandpass bandwidth of the bandpass filter is 0–150 Hz. The following Butterworth amplitude square function is used:

(1) $$\begin{array}{c} {\left|H\left(\omega \right)\right|}^{2}=\frac{1}{1+{\left(\omega /\omega_{c}\right)}^{2n}}=\frac{1}{1+\varepsilon^{2}{\left(\omega /\omega_{p}\right)}^{2n}}. \end{array}$$

In (1), *n* is the order of the filter, and *ω*_*c*_ is the cutoff frequency; that is, the frequency of the amplitude falling to 3 dB. *ω*_*p*_ is the band edge frequency [15].

Then, we use the EEMD method to filter. EEMD is based on EMD, adding the white noise-aided analysis method. The EEMD method considers that the real signal and white noise superposition form the original signal. Each original signal can be obtained by means of EMD to get the IMF component. However, the IMF obtained by EMD alone still contains the effect of white noise. The IMF component directly obtained by the EMD method is a true signal.

(2) $$\begin{array}{c} x_{i}^{\prime }\left(t\right)=\frac{1}{N}\overset{N}{\underset{j=1}{\sum }}x_{ij}\left(t\right). \end{array}$$

In (2), *x*_*ij*_ is the original 1 IMF component with white noise, and *N* is the number of EMD execution times. The white noise per IMF is different, but when the number of EMD executions is sufficient, that is, when the number of IMF is enough, the average of all IMF will be considered to be close to the real result. With more and more tests, the additional noise is considered completely eliminated and only the signal is stable.

**Figure 4:**
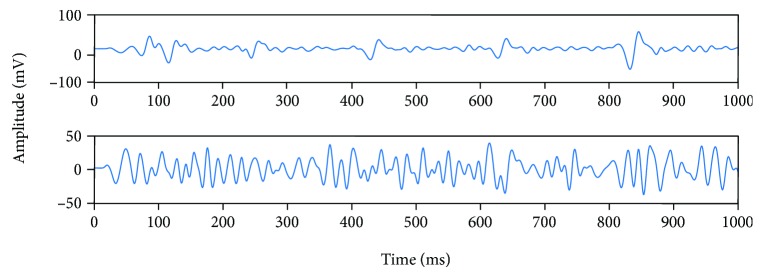
Filtered sEMG signal at a degree of 0.

Figure 4 shows the filtered sEMG signal at a degree of 0. We can see that after the signal preprocessing, the high-frequency component of the sEMG signal is obviously less than the original signal, and the signal is smoother. This is because signal preprocessing has filtered out the high-frequency signal. In this experiment, four modes of electromyography are taken, respectively, but in this paper, only 0 degree signals are cited as examples. After signal preprocessing, feature extraction is needed [16].

### 3.3. Feature Extraction

Wavelet transform (WT) is derived from the localization of short-time Fourier transform, which improves the defect that the frequency cannot change in the transformation process and the size of the window is unchanged from the beginning to the end. It can make the time accuracy change with the frequency. It is an ideal tool for time frequency analysis and processing of the signal. Wavelet transform can highlight the characteristics of the problem, which greatly improves the precision of the frequency time analysis. With the expansion and translation operation wavelet function, the original signal is gradually refined; the time subdivision at high frequency and the frequency subdivision at low frequency are used to solve the problem of Fourier transform and become a new and useful transformation method for signal processing. Therefore, the wavelet energy feature is used as part of the feature space in the design.

Wavelet packet can be considered as a family of functions, where the signal is decomposed in the feature space at a finer level, and the corresponding frequency band is selected for the decomposed signal, which makes each decomposition signal match the original signal spectrum; that is, it has better analysis in time and frequency. Compared with wavelet transform, wavelet packet transform performs better in the decomposition of high frequency, and it is more accurate and does not lose the composition of the original signal. The decomposition diagram is shown in Table 2:

In this method, the highest level (zeroth layer) contains all the frequency band signals, the first layer has 21 nodes, the frequency segment is divided into 21 parts, the *N* layer is divided into 2*N* parts, and there are 2*N* nodes, with each node signal in this node signal band being a signal within the node. Each layer has the maximum amplitude of the node signal, which is the most effective signal in the same layer, which can be considered as representing the effectiveness of the signal on this layer to the maximum extent [17].

The method of wavelet analysis has opened up a new way for the weak signal detection technology, but the key to the feature extraction of the weak signal in the wavelet transform is to determine the threshold of the wavelet coefficients. On the basis of the soft threshold, the concept of wavelet, which reflects the energy distribution characteristic of the signal, has a different wavelet entropy on different decomposition scales, and the threshold of the high-frequency coefficient can be determined adaptively.

Wavelet entropy can accurately locate weak signals in a strong noise environment and achieve effective extraction of the energy's passing signals. Therefore, in the feature space, wavelet entropy is selected as one of the characteristics. Finally, we get a multidimensional feature space.

Finally, the feature space is composed of wavelet energy features and wavelet entropy features.

### 3.4. Pattern Recognition

In the pattern recognition of electromyography, we have made some attempts to classify commonly used algorithms. Finally, through the analysis of results, the random forest can get a good classification effect in the classification algorithm. So in this experiment, the random forest classifier is used to classify. Here are some introductions to the random forest classifier.

In the method of RT, *k* samples are randomly selected from the original training sample set *N* to generate a new set of training samples, and then a random forest is formed by generating *K* classification trees based on the self-help sample set. The classification results of new data are determined by the number of votes cast by the classification tree. The essence is an improvement of the decision tree algorithm, which combines multiple decision trees together. The establishment of each tree depends on an independent sample. Each tree in the forest has the same distribution. The classification error depends on the classification ability of each tree and the correlation between them. Feature selection uses a stochastic method to separate each node, and then compares the errors produced in different situations. The inherent estimation error, classification ability, and correlation can be detected to determine the number of selection features. The classification ability of a single tree may be very small, but after a large number of decision trees are produced randomly, a test sample can choose the most likely classification by the statistics of each tree [18].

### 3.5. Classification Results

**Figure 5:**
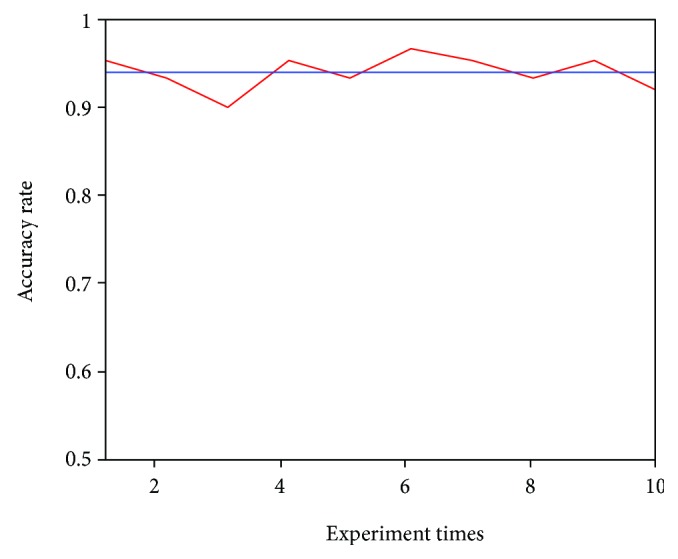
Wavelet packet multilayer decomposition.

We completed the processing, training, and testing of all the EMG signals. The ratio of training samples to test samples is 9 : 1. The random forest classifier is applied to pattern recognition. We have done 10 experiments in all, and the results are shown in Figure 5.

Experimental results show that the classification accuracy of the classifier is about 94%. In the setup of this experiment, we used a 9 : 1 ratio and randomized collected experimental data to classify. This sample includes 0 modes of four actions: 30 degrees, 30 degrees, 60 degrees, and 90 degrees. The model trained by these samples will be used in the classification of the rehabilitation exoskeleton robot. In the actual system operation, we should also take into account the delay time. In some data, the maximum time delay of the patient is 200 ms–400 ms, and the robotic system shows that the average delay is about 300 ms, which is in line with the time delay [19].

## 4. Conclusion

Robot assisted rehabilitation training for hemiplegic patients is a new research hotspot which has attracted more and more attention.

The main structure of the robot is first introduced. The exoskeleton robot overcomes the heavy features of the existing mechanical device. Then, the design for acquiring and processing electromyographic signals for the robotic system is introduced. In this experiment, we use the four action modes of the elbow to recognize the pattern. In the process of signal processing, we take into account the weak and high noise characteristics of the signal, and use EEMD and other methods to preprocess the signal. The feature space of this experiment is composed of wavelet energy features and wavelet entropy features.

The final classification method is based on the random forest method. From the final results, the experiment has achieved good results. In the field of designing rehabilitation robots, one requires its classification accuracy. On the other hand, it also has high requirements for the time delay performance of the algorithm. In the experiment, the average time delay is in line with the actual requirements. Based on the above conclusions, it can be concluded that the robotic system can achieve the actual application requirements in general.

## Figures and Tables

**Table 1:** Range of three degrees of freedom.

3 DOFs of a joint	Elbow joint	Wrist joint	
	Flexion/extension	Flexion/extension	Pronation/supination
Limit angle	0°–150°	0°–150°	−90°–90°
Motion range	0°–120°	0°–120°	−60°–90°

**Table 2:** Wavelet packet multilayer decomposition.

*U*_0_^0^							
*U*_1_^0^				*U*_1_^1^			
*U*_2_^0^		*U*_2_^1^		*U*_2_^2^		*U*_2_^3^	
*U*_3_^0^	*U*_3_^1^	*U*_3_^2^	*U*_3_^3^	*U*_3_^4^	*U*_3_^5^	*U*_3_^6^	*U*_3_^7^

## Data Availability

The data used to support the findings of this study are available from the corresponding author upon request.
